# Phytochemicals in the Prevention and Treatment of SARS-CoV-2—Clinical Evidence

**DOI:** 10.3390/antibiotics11111614

**Published:** 2022-11-13

**Authors:** Katarina Bijelić, Maja Hitl, Nebojša Kladar

**Affiliations:** 1 Department of Pharmacy, Faculty of Medicine, University of Novi Sad, Hajduk Veljkova 3, 21000 Novi Sad, Serbia; 2 Center for Medical and Pharmaceutical Investigation and Quality Control, Faculty of Medicine, University of Novi Sad, Hajduk Veljkova 3, 21000 Novi Sad, Serbia

**Keywords:** phytotherapy, SARS-CoV-2, quercetin, curcumin, baicalin, glycyrrhizin, lycorine, colchicine, black cumin, green propolis

## Abstract

The first case of SARS-CoV-2 infection was reported in December 2019. Due to the rapid spread of the disease and the lack of adequate therapy, the use of plants that have a long history in the treatment of viral infections has often been considered. The aim of this paper is to provide a brief review of the literature on the use of phytochemicals during the new pandemic. An extensive search of published works was performed through platforms Google Scholar, PubMed, Science Direct, Web of Science and Clinicaltrials.gov. Numerous preclinical studies on the use of phytochemicals (quercetin, curcumin, baicalin, kaempferol, resveratrol, glycyrrhizin, lycorine, colchicine) against SARS-CoV-2 have shown that these components can be effective in the prevention and treatment of this infection. Clinical research has proven that the use of black cumin and green propolis as well as quercetin has positive effects. As for other phytochemicals, in addition to preclinical testing which has already been carried out, it would be necessary to conduct clinical tests in order to assert their effectiveness. For those phytochemicals whose clinical efficacy has been proven, it would be necessary to conduct research on a larger number of patients, so that the conclusions are more representative.

## 1. Introduction

In December 2019, the first case of infection with the new coronavirus was reported in Wuhan, China [1]. Due to the similarity with the SARS-CoV virus, this new virus was named SARS-CoV-2 (abbreviated from severe acute respiratory syndrome). Shortly after the appearance of the virus, it spread rapidly throughout the world, and the World Health Organization declared a pandemic on 11 March 2020 [2,3]. By the end of September, 2022, 620,413,942 people were infected and 6,540,871 died worldwide. The countries with the highest number of cases were the United States, India and Brazil (Source: https://www.worldometers.info/coronavirus/, accessed on 26 September 2022).

The SARS-CoV-2 virus belongs to the Coronaviridae family, which includes enveloped, positive-sense single-stranded ribonucleic acid (RNA) viruses [4]. Viruses from this group can infect both humans and animals, and SARS-CoV-2, along with six other viruses, have so far caused infections in humans (SARS-CoV, 229E, HKU1, NL63, OC43 and MERS-CoV) [5]. Although viruses from this family have been known to the scientific public for decades, their clinical significance and epidemic potential were not recognized until the outbreak of SARS (severe acute respiratory syndrome) and MERS (Middle East respiratory syndrome) [6]. The coronavirus disease 2019 (COVID-19) infection spreads rapidly by droplets, most often by sneezing or coughing. The symptoms of infection are mild in the majority of patients; however, in a certain number of people, more severe clinical symptoms appear, which can lead to death [7,8].

Due to the rapid spread of the disease and the lack of adequate therapy, the use of alternative treatment methods was often considered. The use of plants in the therapy of various diseases has been known since ancient times, and they were often used during this pandemic as well. In earlier studies, many plants were shown to have anti-inflammatory, antiviral and antioxidant effects, which made them potential candidates against the SARS-CoV-2 virus [9,10,11,12].

The aim of this paper is to give a brief overview of the literature on the use of phytochemicals and products of natural origin during the pandemic of the new coronavirus. The focus of the paper is given to those phytochemicals previously reported in clinical research conducted on humans. The compounds are chosen on the basis of their clinical effectiveness in combat against the SARS-CoV-2.

## 2. Materials and Methods

### Search of Scientific Literature

The research was conducted by searching the scientific literature via platforms: Google Scholar, PubMed, Science Direct, Web of Science and Clinicaltrials.gov. The keywords “COVID-19” OR “Novel coronavirus” OR “New corona virus” were searched along with “plants” OR “herbals” OR “traditional medicine˝ OR ˝herbal drugs” OR “phytomedicine” OR “phytochemicals” OR “clinical trials” OR “Chinese medicine” OR “Ayurveda” in order to retrieve published articles until September 2022. For the purposes of this research, only publications available in their entirety (not in the form of abstracts) were taken into account; the literature of collected research was also reviewed with the aim of finding additional references. The papers were restricted to those written in the English language. Furthermore, research from the field of clinical research and molecular docking as well as in vitro and in vivo studies were taken into account. Bearing in mind that numerous studies (especially research conducted in vitro and in silico) have been published, this review focused on phytochemicals tested in clinical research in humans, with a short overview of complementary data from preclinical studies.

## 3. SARS-CoV-2

Coronaviruses are positive-sense single-stranded RNA viruses (with symmetric helical nucleocapsid) belonging to the subfamily Orthocronavirinae (family Coronaviridae; order Nidovirales), which contains four genera: Alphacoronavirus, Betacoronavirus, Gammacoronavirus and Deltacoronavirus. SARS-CoV-2 belongs to the Beta genus, which, similar to Alpha, only infects mammals. For the Beta genus, until 2019, six viruses were known to infect humans, of which the most serious infections were caused by SARS-CoV, responsible for the severe acute respiratory syndrome in Guangdong, China, in 2002, and MERS-CoV, responsible for the Middle East respiratory syndrome (MERS) in 2012 in the Kingdom of Saudi Arabia [13,14,15]. Infections caused by these viruses are manifested by a spectrum of symptoms ranging from the common flu to acute respiratory distress syndrome. The most common clinical symptoms of SARS and MERS included fever, cough, myalgia, shortness of breath, as well as upper respiratory tract infections and sore throat. In some of the cases, the disease can progress to pneumonia, further leading to acute respiratory distress syndrome (ARDS) and multiple systemic organ dysfunction (MODS) [6,16].

**Figure 1:**
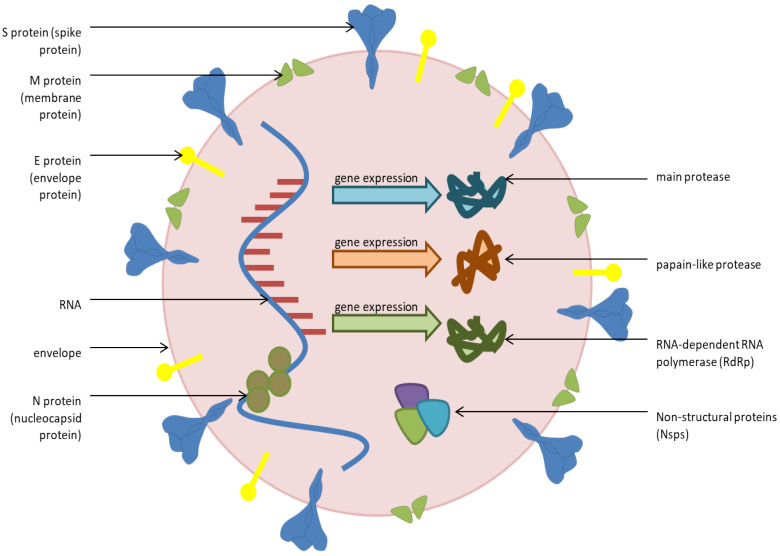
Structure of SARS-CoV-2.

The SARS-CoV-2 genome is made up of about 30,000 nucleotides and encodes twenty different proteins, including four main structural proteins: S—spike; E—envelope; M—membrane; and N—nucleocapsid (Figure 1). In addition, the virus also has non-structural proteins such as RNA-dependent RNA polymerase (RdRp), coronavirus main protease (3Clpro) and papin-like protease (PL-pro), which have an important role in replication [17,18,19,20,21,22]. The spike protein has a receptor-binding domain (RBD) that is responsible for the virus binding to the target angiotensin-converting enzyme 2 (ACE2)- key receptor for viruses entering into human cells. In order for the virus to complete entry into the cell, it is necessary that the complex of S protein–ACE2 is cleaved by a transmembrane serine protease (TMPRSS2), after which fusion of the virus and the host cell membrane begins. The virion then releases RNA into the host cell (which becomes uncoated), where it undergoes replication and translation, leading to further spread of viral particles in the human body [14,23,24,25]. The epithelium of the upper respiratory tract represents the first point of attack of the virus, while other organs that express ACE2 can also be the target of attack, such as the heart, kidneys and small intestine [24,26].

The main route of infection transmission from person to person is via respiratory droplets (sneezing, coughing) [27].

The first clinical signs of infection usually appear after 5.2 days (incubation time) on average, while in a certain number of people, the infection may pass without any symptoms [28]. The symptoms that occur are mostly non-specific such as fever, sore throat, cough, fatigue, myalgia and, less often, headache, sputum production, diarrhea and dyspnea [29]. Some patients may develop severe clinical symptoms, which is caused by a cytokine storm (release of high amounts of pro-inflammatory cytokines such as: interleukin 2 (IL-2), interleukin 7 (IL-7), interleukin 10 (IL-10), granulocyte colony-stimulating factor (G-CSF), interferon-gamma-induced protein 10 (IP-10), monocyte chemoattractant protein-1 (MCP-1), macrophage inflammatory protein-1α (MIP-1A) and tumor necrosis factor-alpha (TNFα)) [30]. The consequences of cytokine storm include viral sepsis, inflammatory lung damage including pneumonitis, acute respiratory distress syndrome, shock, multi-organ failure (acute kidney damage, liver damage, myocarditis) and in the worst case, death [31,32].

## 4. Therapeutic Potential of Medicinal Plants

Complementary and alternative medicine offers a wide range of treatment methods. Phytotherapy is one of the most well-known and used in the treatment of various diseases [33]. According to the World Health Organization (WHO), 80% of the population in developing countries rely on the use of traditional plants in therapy [34,35]. Natural products and their derivatives can potentially be used in the therapy of numerous viral infections, and this use has been known since ancient times, especially the use of plants from Chinese traditional medicine and Indian Ayurvedic medicine. [36,37]. Since the SARS-CoV-2 virus epidemic progressed rapidly, and there was no adequate therapy, several countries have analyzed the role of medicinal plants (both in their original form and in the form of various preparations) in COVID-19 therapy [38]. Bearing in mind that numerous plants have a long tradition of use in respiratory and infectious diseases, the research often turns to plants that have already been proven to have an antiviral effect (against human immunodeficiency virus (HIV), SARS, MERS, influenza), as well as to immunostimulating plants for the prevention of the disease [39,40]. *Curcuma longa* Zingiberaceae, *Glycyrrhiza glabra*, Fabaceae, *Artemisia annua*, Asteraceae, *Scutellaria baicalensi*, Lamiaceae, *Lycoris radiata*, Amaryllidaceae as well as various mixtures of plants used in Chinese traditional medicine are often considered as potential agents against SARS-CoV-2 [41]. Each of the potential effects of the plants has been confirmed either by in silico analysis, or by in vitro and in vivo analysis, but rarely in clinical studies.

## 5. Phytochemicals as Potential Therapeutics in SARS-CoV-2 Virus Infection

Plants have a long tradition of use, but modern research aims to determine which compounds are responsible for the pharmacological effects. Most often, the activity is attributed to the most represented compound (in percentage), but the synergistic effect of a large number of compounds present in the plant material cannot be ignored. Precisely through chemical analyses, numerous specific compounds in plants were discovered, which, due to their pharmacological activity, have also found a place in clinical research (including research on patients with COVID-19 infection) [42].

### 5.1. Quercetin

Quercetin is a flavonoid compound which can be found in most fruits (citrus fruits, apples), vegetables (onions, parsley), seeds and grapes [18,43]. Dietary supplements containing this compound are widely used as immunostimulant products. It has an excellent safety profile and has been declared by the United States Food and Drug Administration (FDA) as a safe compound (GRAS status—generally recognized as safe) in dietary products for human use [44,45].

Quercetin shows anti-inflammatory, antioxidant, antiviral and immune-protective effects, which triggered its evaluation in numerous studies that analyzed the potential use of phytochemicals against SARS-CoV-2 [46,47,48]. The antioxidant potential of quercetin was proven by numerous in vitro and in vivo studies [49,50]. It acts as a scavenger of free radicals, inhibits lipid peroxidation and, thus, protects the body from reactive oxygen species. In addition, quercetin leads to the inhibition of the release of pro-inflammatory cytokines, inhibiting their LPS (lipopolysaccharide)-stimulated release, which plays an important role in preventing the onset of a cytokine storm [47,51]. The antiviral effect of quercetin was confirmed against several respiratory viruses such as influenza virus, parainfluenza virus, respiratory syncytial virus and adenovirus [44,52].

In silico studies have indicated binding of quercetin to the protein targets of SARS-CoV-2 (including the S protein, which is of primary interest for the entry of the virus into the cell, and the main protease, which plays a role in replication) [45,53]. 

So far, nine clinical studies have been completed that have considered the use of quercetin in treatment of COVID-19 [54]. One of them considered the use of curcumin, quercetin and vitamin D3 as additional therapy in the early phase of COVID-19 infection. It was a pilot open-label, randomized controlled trial conducted in Pakistan. The results of this research showed that the use of this combination of components leads to a reduction in acute symptoms, modulation of the hyperinflammatory response and that, as an adjunctive therapy, it can contribute to a faster recovery of patients. However, due to the small number of patients, additional studies are necessary [55]. Furthermore, a randomized clinical study was conducted in Iran where the effectiveness of quercetin in combination with antiviral therapy was investigated. The results indicated that quercetin reduces the period of hospitalization and reduces the level of C-reactive protein (CRP) and some enzymes such as lactate dehydrogenase (LDH) and alkaline phosphatase (ALP). However, there was no observed difference in mortality, duration of ICU admission and the number of ICU-admitted cases [56].

### 5.2. Curcumin

Curcumin is the most famous polyphenolic compound isolated from the turmeric plant (*Curcuma longa*, Zingiberaceae). The extract of this plant has a long history of use in Ayurvedic medicine for respiratory problems, such as runny nose, cough and sinusitis. This compound is known to have antioxidant, anti-inflammatory and anti-diabetic properties as demonstrated by in vitro and in vivo studies [57,58,59].

Several studies have tested the effect of curcumin against influenza viruses, herpes virus, respiratory syncytial virus, where it has been proven that this substance has an antiviral effect, as well as modulating the immune response and inhibiting the cytokine storm. All these effects highlighted curcumin as a potential component against COVID-19 [57,60]. In silico studies have shown that curcumin has an affinity for the receptor-binding domain of the SARS-CoV-2 protein [61]. It has also been shown that it reduces the expression of the ACE2 receptor [62]. The clinical application of nano-curcumin for oral administration (made to improve bioavailability) was shown to reduce levels of pro-inflammatory cytokines, shorten hospital stay and increase blood oxygen levels, compared to placebo. However, the clinical significance of the recorded results should be confirmed on a larger group of patients [63].

### 5.3. Baicalin

Baicalin is a flavonoid compound extracted from *Scutellaria baicalensi*, Lamiaceae (Huang Qin), a plant widely used in traditional Chinese medicine. It can also be found in other species of the genus Scutellaria (*Scutellaria planipes*, *Scutellaria rehderiana* and *Scutellaria scandens*) as well as in *Oroxylum indicum*, Bignoniaceae, *Sophora tonkinensis*, Fabaceae, *Angelica sinensis* and Apiaceae [64]. Baicalin has a wide range of actions such as antioxidant, anti-inflammatory and antiviral properties [38,65]. In silico studies have shown that this compound inhibits the three most important proteases of the SARS-CoV-2 virus: main, papain-like and RNA-dependent polymerase. It also showed inhibition of these proteases in in vitro studies [66]. Since baicalin was previously recognized for its anti-SARS activity, it was widely considered as a potential phytotherapeutic for SARS-CoV-2 [67]. Furthermore, in H5N1 virus infection, it was shown that baicalin lowers the levels of IL-6 and IL-8, which would also be useful in COVID-19 treatment [18].

Therefore, due to its action, low toxicity and long-demonstrated history of traditional use, it would be desirable to test the effectiveness of this compound through clinical trials. Although low oral bioavailability may represent a problem, it could be successfully solved by design of the appropriate pharmaceutical formulations [65,68].

### 5.4. Kaempferol

Kaempferol is a flavonoid found in a large number of edible plants (e.g., tea, broccoli, cabbage, kale, beans, endive, leek, tomato, strawberries and grapes). This compound has shown a wide range of pharmacological activities such as anti-inflammatory, antiviral, anti-cancer and anti-bacterial properties [69]. In vitro studies have demonstrated its activity against H1N1 and H9N2 viruses. Activity against herpes simplex virus and HIV virus has also been recorded [18,70,71]. Kaempferol was shown to have a high degree of binding to the main protease of SARS-COV-2, which could potentially disrupt viral replication. It was also shown that it can bind to the ACE2–S complex, which could prevent the internalization of the virus into the host cell [72,73]. Moreover, kaempferol lowers pro-inflammatory cytokines, which play a role in the onset of cytokine storm [74]. However, in order to confirm the antiviral effect of this component, it is necessary to conduct more clinical trials since kaempferol has a low bioavailability. It should be determined whether it is possible to achieve concentrations that would lead to the manifestation of the desired pharmacological activity through oral consumption [69,75].

### 5.5. Resveratrol

Resveratrol is a compound that belongs to the group of polyphenolic compounds, more precisely, to the group of stilbenoids [76]. It can be found in *Vitis vinifera* (Vitaceae), *Morus nigra* (Moraceae), some other grapes and in peanuts [75]. Resveratrol has been known for its wide pharmacological activity and is the most studied compound from the stilbenoid group [77]. Previous research has shown that this compound has antiviral, antioxidant, anti-inflammatory and anti-tumor properties [74,78]. So far, numerous in vitro and in vivo studies have proven that resveratrol acts on various respiratory viruses such as influenza and respiratory syncytial virus and also on coronaviruses such as SARS and MERS [77,79,80,81]. It reduces the expression of the nucleocapsid protein and inhibits the replication of the MERS-CoV virus [77]. Further, in an in vitro study, it was shown to have an effect against the SARS-CoV-1 virus [76]. Some in silico studies have shown that resveratrol has a high degree of binding to the ACE2 complex (compared to other stilbenoids) [82]. It has also been proven to inhibit the proliferation of SARS-CoV-2 on mammalian cells cultures, which distinguishes it as a potential phytotherapeutic for the treatment of this viral infection [83]. Additionally, resveratrol inhibits the effect of proinflammatory cytokines such as IFN-γ (interferon gamma), TNF-α and IL-1β, which play a major role in the onset of cytokine storm in COVID-19 [78]. However, one of the problems with the use of resveratrol is that it has low oral bioavailability, and in order to achieve optimal concentrations in the blood and exhibit antiviral and anti-inflammatory activity, it is necessary to develop a suitable pharmaceutical formulation [75,84].

### 5.6. Glycyrrhizin

Glycyrrhizin, a triterpene saponin, is one of the main components isolated from *Glycyrrhiza glabra*, Fabaceae, a plant with a long history of use [85]. On the market, it can often be found in the form of dietary supplements. *Glycyrrhiza glabra* and its derivatives are generally recognized as safe by the FDA (GRAS) [85,86]. Glycyrrhizin has a wide range of antiviral activity (HIV, porcine reproductive and respiratory syndrome virus, human respiratory syncytial virus, influenza viruses, herpes viruses, hepatitis B and C) [77,87]. During the SARS CoV-1 virus epidemic, the effectiveness of glycyrrhizin against this virus was investigated. In a study on Vero cells, it was proven that glycyrrhizin inhibits the replication of this virus (FFM1 and FFM2—two clinical isolates of SARS-associated coronavirus) and reduces the penetration of the virus into host cells. Due to its proven efficacy against SARS-CoV, it was a candidate for testing against SARS-CoV-2 [88,89]. The docking studies indicated a strong binding of glycyrrhizin to ACE2, as well a reduction in the expression of the transmembrane serine protease [65,87]. Furthermore, it has been proven to reduce the release of pro-inflammatory cytokines such as TNFα, IL6 and IL1 β, which can have effects on preventing the development of more severe clinical symptoms [86,88]. However, glycyrrhizin is rapidly metabolized in human body, so an effective concentration in the serum to suppress virus replication was not successfully achieved. One of the approaches suggested, with the aim to improve its bioavailability, was to make modifications in the structure (formation of amide). This way, a better effect against viruses was achieved, but the toxicity was also increased [90]. Glycyrrhizin has proven to be an extremely potent component, and it would be desirable to examine its effects through clinical trials.

### 5.7. Lycorine

Lycorine is a phenanthridine alkaloid isolated from *Lycoris radiata*, Amaryllidaceae, a well-known plant in traditional Chinese medicine [18]. It has also been identified in various genera of the Amaryllidaceae family such as *Ammocharis*, *Boophane*, *Brunsvigia*, *Crinum*, *Galanthus*, *Haemanthus*, *Hippeastrum*, *Hymenocallis*, *Leucojum*, *Lycoris*, *Narcissus*, *Sternbergia* and *Zephyranthes* [91]. This alkaloid has a wide range of activities such as antiviral, anti-bacterial, anti-inflammatory, anti-cancer properties, etc. [92]. In vitro studies have shown that lycorine is effective against the SARS-CoV-1 virus, as well as several other coronaviruses (including MERS). It also showed anti-SARS-CoV-2 activity in a test with Vero cells, where its effectiveness was on par with remdesivir (an antiviral drug). Molecular docking showed that lycorine binds more strongly than remdesivir to the RdRp of the SARS-CoV-2 virus and, thus, could inhibit virus replication. Certainly, more research is needed to determine the exact mechanism of action of this substance [93,94]. The advantages of this alkaloid are its low toxicity and minimal side effects [75].

### 5.8. Colchicine

Colchicine is a tricyclic, liposoluble alkaloid obtained from the plant *Colchicum autumnale*, Liliaceae, whose use has been known since ancient times [95,96]. Oral colchicine is FDA-approved for the treatment of gout and Mediterranean family fever. Because of its anti-inflammatory and antiviral effects, this compound has been the target of numerous studies, including several clinical trials (clinicaltrals.gov) [97]. The anti-inflammatory effects of colchicine are based on the inhibition of leukocytes (monocytes and neutrophils). In addition, it prevents their adhesion to endothelial cells (the first step in the pathogenesis of inflammation) and, thus, prevents their migration into the inflamed tissue. Colchicine also has the ability to modulate the production of pro-inflammatory cytokines such as IL-1, IL-6 and TNFα [97,98]. As colchicine binds tubulin and has a harmful effect on the polymerization of microtubules, in theory, it could prevent the replication and transcription of coronaviruses (including SARS-CoV-2), because their entry into the cell requires interaction with microtubules and the cytoskeleton [99,100]. An extensive double-blind, placebo-controlled clinical study on the use of colchicine in COVID-19 therapy, which included approximately 4500 patients, was conducted and coordinated by the Montreal Heart Institute. Although expectations were high for this study, it was unfortunately terminated (as stated on the clinicaltrials.gov website, due to human, logistical and budgetary reasons) [101].

### 5.9. Artemisinin

Artemisinin is a sesquiterpene compound that is isolated from the plant *Artemisia annua*, Asteraceae. The use of this plant in traditional medicine has been present for centuries both on the Asian and African continents. It was used to treat malaria, as well as related fevers [41,102,103]. Artemisinins (dihydroartemisinin, artemether–lumefantrine, artesunate, arteether, arteannuin B and artemisone) are a group of artemisinin-related substances developed to treat malaria [104]. It has been reported that they possess broad antiviral as well as anticarcinogenic and immunomodulatory activities. Artesunate has been shown to act against DNA and RNA viruses, including human cytomegalovirus (HCMV), human herpes simplex virus (HSV), hepatitis B virus (HBV), hepatitis C virus (HCV) and human immunodeficiency virus (HIV), while dihydroartemisinin has been shown to work against the Zika virus. Further, during the SARS virus epidemic, *Artemisia annua* extract showed inhibitory activity, which made these compounds potential candidates in the therapy of the new SARS-CoV-2 virus [41,104].

In vitro studies on Vero 6 cells showed that artemisins inhibit the replication of the SARS-CoV-2 virus and that arteannuin B completely blocks the nucleocapsid protein of this virus [104,105]. Both the plant extract and artemisinins have been shown to be anti-inflammatory agents, reducing the levels of cytokines such as IL-1, IL-6 and TNFα, which would have beneficial effects on cytokine storm [103,105,106].

A small clinical study was conducted in China in 2020 involving 41 patients, who were divided into two groups, one receiving artesunate and piperaquine (available antimalarial drugs), and the other being a control group. In the treated group, in patients with mild to moderate symptoms, a shorter retention of the virus in the body was demonstrated. However, some patients experienced side effects such as prolongation of the QT interval, which could be a limiting factor for the use of this therapy. Due to the small number of patients included in this study, more research is needed [107].

**Figure 2:**
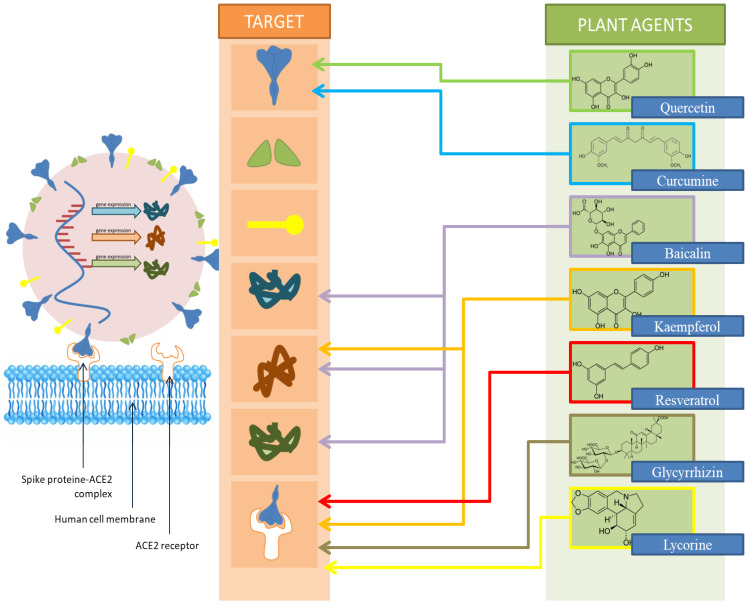
Schematic representation of the site of action of the listed phytochemicals.

A schematic representation of the site of action of the mentioned compounds is shown in Figure 2.

Numerous other compounds isolated from medicinal plants and fruits and vegetables are found by in silico studies to be effective against SARS-CoV-2. Some of them are presented in Table 1, together with biological sources and the targeted structure in coronavirus.

## 6. The Effectiveness of Phytotherapeutics—Proven by Clinical Research

A multicenter open-label, randomized, controlled clinical trial was conducted in five centers in Iran during 2020. Plants recognized by Persian traditional medicine were used for the preparation of phytotherapeutics. The first drug consisted of capsules containing lyophilized water–ethanol extract of the rhizome of *Rheum palmatum*, Polygonaceae, root of *G. glabra*, and fruit peel of *Punica granatum*, Punicaceae. The second remedy contained 500 mg of powdered black cumin (*Nigella sativa*, Ranunculaceae). The third included a decoction made from *Matricaria chamomilla*, Asteraceae, *Zataria multiflora*, Lamiaceae, *G. glabra*, *Ziziphus jujuba*, Rhamnaceae, *Ficus carica*, Moraceae, *Urtica dioica*, Urticaceae, *Althaea officinalis*, Malvaceae and *Nepeta bracteata*, Lamiaceae. Each day, 900 mL of the decoction was made, which was divided into three daily portions (300 mL) [116]. A total of 358 patients were included in the study, 174 received conventional therapy according to the clinical guidelines of the Iranian Ministry of Health (azithromycin, hydroxychloroquine, lopinavir/ritonavir), and 184 received herbal medicines in addition to standard therapy (decoction every 8 h and capsules every 12 h) for 7 days. The research results have shown that the use of supportive herbal therapy reduces the length of hospital stay and leads to relief of symptoms such as runny nose, cough, fever and myalgia (accelerates clinical improvement) [116].

Another clinical trial was conducted in Saudi Arabia from May to September 2020. It was a prospective, two-arm, randomized, open-label study that examined the use of black cumin oil in the treatment of adults with mild symptoms of COVID-19. A little less than 200 patients participated in the study and were divided into two groups: those who received standard therapy and those who received standard therapy with *Nigella sativa* oil (500 mg capsules, 2 times a day after meals, for 10 days). The results of the study showed that the average recovery time was shorter in the group that received black cumin oil. Additionally, for a period of 14 days, the percentage of recovered patients was higher in the group that received this phytotherapeutic. A shorter duration of symptoms such as anosmia, runny nose and loss of appetite was observed in the treated group. It is also stated that it is necessary to conduct more randomized clinical studies, which could include the measurement of some laboratory parameters that would be better indicators of the effect of this herb [117].

Another multicenter, placebo-controlled, randomized trial observing the use of black cumin (in combination with honey) was conducted in four health facilities in Pakistan. About three hundred patients participated in the study and were divided into two groups according to the severity of symptoms (mild to moderate, and another group of patients with more severe symptoms). Within each group, half of the patients received standard therapy (antipyretics, antibiotics, steroids, anticoagulants, supplemental oxygen), and the other half received honey and encapsulated black cumin seeds for 13 days in addition to standard therapy. The results show that in the groups that received honey and black cumin (both those with milder and those with more severe symptoms), the time for symptom relief was statistically significantly shorter compared to the group that did not receive these supplements. Furthermore, the period of clearing from the virus was shorter and a better clinical score was reached faster on the sixth day. Mean oxygen saturation >90% in severe cases was achieved 6 days earlier in the intervention group. In severe cases, the mortality rate was four times lower in the group that received phytotherapeutics. Another good side of this therapy is that it is cheap, easily available and side effects are not recorded [118].

A single-center, open-label, randomized clinical study was conducted in northern Brazil during 2020. Patients who were included in the study (125 patients in total) were divided into three groups: the first, which received only standard therapy for SARS-CoV-2; and the second and third, which, along with standard therapy, received 400 mg and 800 mg of dehydrated standardized green propolis extract for 7 days. Both propolis-supplemented groups were associated with a statistically significant shorter hospital stay compared to the non-propolis-supplemented group. The time spent on oxygen was not statistically significantly different between the groups. Further, patients who received a higher dose of propolis had significantly less acute kidney injury compared to the control group, which is of high significance, since acute kidney injury is one of the most serious consequences of SARS-CoV-2 infection. All the positive effects of propolis were probably related to the presence of polyphenolic compounds such as quercetin and kaempferol [119].

An interesting multicenter, prospective, double-blind, randomized, placebo-controlled study was conducted in two Indian hospitals (600 patients participated, divided into two groups, treated and placebo). The effectiveness of nasal spray (which contained, among other constituents, ginger oil, eucalyptus oil, basil oil and clove oil) in the prevention of SARS-CoV-2 infection was tested. The spray was applied three times a day for 45 days. It has been shown that the use of this spray significantly reduces infections with the mentioned virus in healthcare workers, with 62% less compared to placebo. These effects could be related to the wide range of effects of the essential oils included in this spray [120,121].

One clinical trial involving 80 patients with COVID-19 was conducted in Beijing, China. All patients received symptomatic and supportive treatment, and in addition, 44 of them received Jinhua Qinggan granules. These granules contain a mixture of components: *Forsythia suspensa*, Oleaceae, *Lonicera japonica*, Caprifoliaceae, *Ephedra sinica*, Ephedraceae, *Prunus sibirica*, Rosaceae, *l-Menthol*, *Glycyrrhiza glabra*, *Scutellaria baicalensis*, *Fritillaria thunbergii*, Liliaceae, *Anemarrhena asphodeloides*, Asparagaceae, *Arctium lappa*, Asteraceae and *Artemisia annua*, and are approved for the treatment of influenza virus. In the treated group, it was shown that the application of these granules can significantly reduce the duration of virus nucleic acid detection, as well as lead to a faster recovery from pneumonia, without the appearance of any side effects, which is why this combination of substances should continue to be investigated [41,122,123].

Another small clinical study conducted in China in 2020 examined the use of Xuebijing, a Chinese traditional injection whose main components are *Carthami flos* (*Carthamus tinctorius*, Asteraceae), *Paeoniae rubra radix (Paeonia lactiflora*, Paeoniaceae), *Chuanxiong rhizoma* (*Ligusticum chuanxiong*, Apiaceae), *Salviae miltiorrhizae radix et rhizoma* (*Salvia miltiorrhiza*, Lamiaceae) and *Angelicae sinensis radix* (*Angelica sinensis*, Apiaceae). Sixty patients, divided into three groups, were included in this study. The first group received standard therapy, the second received 50 mL Xuebijing injections twice a day, and the third received 100 mL Xuebijing injections, also twice a day. After treatment, the number of white blood cells (WBC) and lymphocytes was increased in all groups, while C-reactive protein and erythrocyte sedimentation rate (ESR) decreased. When comparing the two groups that received Xuebijing, the group that received 100 mL showed a statistically significant increase in WBC as well as a decrease in CRP and ESR. Furthermore, after the treatment, the APACHE II score decreased in all three groups, while in the group that received 100 mL of Xuebijing, it was significantly lower compared to the one that received 50 mL. More research is needed for determining the exact mechanisms of action of this formulation [41,124].

## 7. Future Prospects

All these herbal compounds have been investigated as adjuvant therapies to the conventional one prescribed for the treatment of SARS-CoV-2. As of the end of September, none of the herbal therapies listed in this paper have been approved by the FDA or any other regulatory agency. Although numerous studies have shown the effectiveness of the mentioned phytochemicals, further research which demonstrates effectiveness and safety at the same time is mandatory. Numerous works of research are still ongoing in search of the most effective phytochemical or a combination of them that would be the most effective adjuvant(s) in treating this viral infection.

Some future research that would be based on confirming the activity of herbal components on a larger number of people would be the basis for the approval of herbal therapy by regulatory agencies. Including phytotherapy in conventional protocols for treatment of COVID-19 would widen the range of available therapies, making the treatment of this newly discovered virus easier, innovative and safer.

## 8. Conclusions

Plants are a rich source of a large number of phytochemicals, which possess a wide spectrum of pharmacological activity. Preclinical studies often suggest wide spectra of potential anti-SARS-CoV-2 activities. However, in order to facilitate clinical application, appropriate studies on patients have to be conducted in order to confirm the efficacy and safety of application of the specific agent. The currently available data suggest that, although some compounds of natural origin have been evaluated in clinical studies, these generally included a small number of patients and the results obtained have to be considered with caution (most of the patients participating in the studies mentioned above, 600 of them, were included in the nasal spray efficacy trial, which is not a representative sample compared to the 60 million patients affected by the virus). Furthermore, there is a scientific need to repeat these studies in future on a larger number of patients, so that the conclusions obtained are of higher relevance. Regarding the treatment of COVID-19, the available data suggest that so far, the most promising agents of natural origin that can positively affect the symptoms and outcomes of the disease are quercetin, glycyrrhizin, resveratrol, kaempferol as well as thymoquinone (an active component from black cumin). Generally, the advantage of phytochemicals compared to conventional drugs is that those with a long-demonstrated history of use generally have no side effects (and some have GRAS status) and people have no fear when consuming these drugs because they consider them natural. However, problems of low bioavailability are often encountered when using phytotherapeutics, which gives space for research in the field of pharmaceutical technology (how to find the appropriate drug formulation).

## Figures and Tables

**Table 1:** Phytochemicals as potential agents against SARS-CoV-2 (proved by molecular docking studies).

Active Compound	Source	Structure of SARS-CoV-2 Considered Targeted by Given Phytochemicals	References
myrcetin	in most fruits, vegetables, tea and wine	SARS-CoV-2 3Clpro	[108]
apigenin	in many fruits and vegetables, in plants such as *Petroselinum crispum*, Apiaceae and *Matricaria chamomila*, Asteraceae	SARS-CoV-2 3Clpro	[109]
luteolin	vegetables and fruits such as celery, parsley, broccoli, onion leaves, carrots, peppers, cabbages andapple	SARS-CoV-2 3Clpro, PL-pro and ACE2	[110]
abyssinone II	Chinese medicinal plant *Broussonetia papyrifera*, Moraceae	SARS-CoV-2 3Clpro, PL-pro and ACE2	[110]
green tea polyphenols	green tea	SARS-CoV-2 3Clpro	[111]
gingerol	*Zingiber officinale*, Zingiberaceae	SARS-CoV-2 3Clpro	[112]
cannabidiol	*Cannabis sativa*, Cannabaceae	SARS-CoV-2 3Clpro, PL-pro and ACE2	[113]
allicin	*Allium sativum*, Amaryllidaceae	SARS-CoV-2 3Clpro	[114]
withanoside V and somniferine	*Withania somnifera*, Solanaceae (Ashwagandha)	SARS-CoV-2 3Clpro	[115]
tinocordiside	*Tinospora cordifolia*, Menispermaceae	SARS-CoV-2 3Clpro	[115]

## Data Availability

Not applicable.
