# Correction: Bijelić et al. Phytochemicals in the Prevention and Treatment of SARS-CoV-2—Clinical Evidence. *Antibiotics* 2022, *11*, 1614

**DOI:** 10.3390/antibiotics14080789

**Published:** 2025-08-04

**Authors:** Katarina Bijelić, Maja Hitl, Nebojša Kladar

**Affiliations:** 1 Department of Pharmacy, Faculty of Medicine, University of Novi Sad, Hajduk Veljkova 3, 21000 Novi Sad, Serbia; maja.bekut@mf.uns.ac.rs (M.H.); nebojsa.kladar@mf.uns.ac.rs (N.K.); 2 Center for Medical and Pharmaceutical Investigation and Quality Control, Faculty of Medicine, University of Novi Sad, Hajduk Veljkova 3, 21000 Novi Sad, Serbia

## Text Correction

There was an error in the original publication [1].

1. A correction has been made to Section 6. The Effectiveness of Phytotherapeutics—Proven by Clinical Research, Paragraph Number 5 (Because of the retracted reference, the entire paragraph should be deleted).
2. In paragraphs 6 and 7, in Section 6. the references need to be renumbered due to the removal of the previous two.
3. A correction has been made to Section 8. Conclusions (The sentence in parentheses (most of the patients participating in the studies mentioned above, 600 of them, were included in the nasal spray efficacy trial, which is not a representative sample compared to the 60 million patients affected by the virus) refers to a retracted reference and should be deleted).

Corrected paragraphs:

Section 6. The Effectiveness of Phytotherapeutics—Proven by Clinical Research, Paragraphs Number 6 and 7:

One clinical trial involving 80 patients with COVID-19 was conducted in Beijing, China. All patients received symptomatic and supportive treatment, and in addition, 44 of them received Jinhua Qinggan granules. These granules contain a mixture of components: *Forsythia suspensa*, Oleaceae, *Lonicera japonica*, Caprifoliaceae, *Ephedra sinica*, Ephedraceae, *Prunus sibirica*, Rosaceae, *l-Menthol*, *Glycyrrhiza glabra*, *Scutellaria baicalensis*, *Fritillaria thunbergii*, Liliaceae, *Anemarrhena asphodeloides*, Asparagaceae, *Arctium lappa*, Asteraceae and *Artemisia annua*, and are approved for the treatment of influenza virus. In the treated group, it was shown that the application of these granules can significantly reduce the duration of virus nucleic acid detection, as well as lead to a faster recovery from pneumonia, without the appearance of any side effects, which is why this combination of substances should continue to be investigated [41,120,121].

Another small clinical study conducted in China in 2020 examined the use of Xuebijing, a Chinese traditional injection whose main components are *Carthami flos* (*Carthamus tinctorius*, Asteraceae), *Paeoniae rubra radix* (*Paeonia lactiflora*, Paeoniaceae), *Chuanxiong rhizoma* (*Ligusticum chuanxiong*, Apiaceae), *Salviae miltiorrhizae radix et rhizoma* (*Salvia miltiorrhiza*, Lamiaceae) and *Angelicae sinensis radix* (*Angelica sinensis*, Apiaceae). Sixty patients, divided into three groups, were included in this study. The first group received standard therapy, the second received 50 mL Xuebijing injections twice a day, and the third received 100 mL Xuebijing injections, also twice a day. After treatment, the number of white blood cells (WBC) and lymphocytes was increased in all groups, while C-reactive protein and erythrocyte sedimentation rate (ESR) decreased. When comparing the two groups that received Xuebijing, the group that received 100 mL showed a statistically significant increase in WBC as well as a decrease in CRP and ESR. Furthermore, after the treatment, the APACHE II score decreased in all three groups, while in the group that received 100 mL of Xuebijing, it was significantly lower compared to the one that received 50 mL. More research is needed for determining the exact mechanisms of action of this formulation [41,122].

8. Conclusions:

Plants are a rich source of a large number of phytochemicals, which possess a wide spectrum of pharmacological activity. Preclinical studies often suggest wide spectra of potential anti-SARS-CoV-2 activities. However, in order to facilitate clinical application, appropriate studies on patients have to be conducted in order to confirm the efficacy and safety of application of the specific agent. The currently available data suggest that, although some compounds of natural origin have been evaluated in clinical studies, these generally included a small number of patients and the results obtained have to be considered with caution. Furthermore, there is a scientific need to repeat these studies in future on a larger number of patients, so that the conclusions obtained are of higher relevance. Regarding the treatment of COVID-19, the available data suggest that so far, the most promising agents of natural origin that can positively affect the symptoms and outcomes of the disease are quercetin, glycyrrhizin, resveratrol, kaempferol as well as thymoquinone (an active component from black cumin). Generally, the advantage of phytochemicals compared to conventional drugs is that those with a long-demonstrated history of use generally have no side effects (and some have GRAS status) and people have no fear when consuming these drugs because they consider them natural. However, problems of low bioavailability are often encountered when using phytotherapeutics, which gives space for research in the field of pharmaceutical technology (how to find the appropriate drug formulation).

With this correction, the order of some references has been adjusted accordingly. The authors state that the scientific conclusions are unaffected. This correction was approved by the Academic Editor. The original publication has also been updated.
